# Constructing a prognostic risk model for Alzheimer’s disease based on ferroptosis

**DOI:** 10.3389/fnagi.2023.1168840

**Published:** 2023-04-27

**Authors:** Xiao-Li Wang, Rui-Qing Zhai, Zhi-Ming Li, Hong-Qiu Li, Ya-Ting Lei, Fang-Fang Zhao, Xiao-Xiao Hao, Sheng-Yuan Wang, Yong-Hui Wu

**Affiliations:** ^1^Department of Occupational Health, Public Health College, Harbin Medical University, Harbin, China; ^2^College of Bioinformatics Science and Technology, Harbin Medical University, Harbin, China

**Keywords:** Alzheimer’s disease, ferroptosis, PC12, Aβ_1–42_, prognostic risk model

## Abstract

**Introduction:**

The aim of this study is to establish a prognostic risk model based on ferroptosis to prognosticate the severity of Alzheimer’s disease (AD) through gene expression changes.

**Methods:**

The GSE138260 dataset was initially downloaded from the Gene expression Omnibus database. The ssGSEA algorithm was used to evaluate the immune infiltration of 28 kinds of immune cells in 36 samples. The up-regulated immune cells were divided into Cluster 1 group and Cluster 2 group, and the differences were analyzed. The LASSO regression analysis was used to establish the optimal scoring model. Cell Counting Kit-8 and Real Time Quantitative PCR were used to verify the effect of different concentrations of Aβ_1–42_ on the expression profile of representative genes *in vitro*.

**Results:**

Based on the differential expression analysis, there were 14 up-regulated genes and 18 down-regulated genes between the control group and Cluster 1 group. Cluster 1 and Cluster 2 groups were differentially analyzed, and 50 up-regulated genes and 101 down-regulated genes were obtained. Finally, nine common differential genes were selected to establish the optimal scoring model. *In vitro*, CCK-8 experiments showed that the survival rate of cells decreased significantly with the increase of Aβ_1–42_ concentration compared with the control group. Moreover, RT-qPCR showed that with the increase of Aβ_1–42_ concentration, the expression of POR decreased first and then increased; RUFY3 was firstly increased and then decreased.

**Discussion:**

The establishment of this research model can help clinicians make decisions on the severity of AD, thus providing better guidance for the clinical treatment of Alzheimer’s disease.

## Highlights

- The international advanced ssGSEA algorithm was used to analyze the difference of immune infiltration degree of GEO database samples.
- A prognostic scoring formula for Alzheimer’s disease was established based on ferroptosis for the first time.
- A prognostic model of Alzheimer’s disease was established by combining *in vitro* experiments with bioinformatics.

## 1. Introduction

Alzheimer’s disease (AD) is a neurodegenerative disease that has a significant impact on the quality of life of patients and their families and is one of the main causes of dementia (Scheltens et al., 2016). Currently, more than 30 million people worldwide suffer from AD, and its prevalence is expected to triple by 2050, mainly due to the aging of the population. As one of the costliest chronic diseases, AD is not only a true global epidemic, but also a huge economic burden in modern society (Pereira et al., 2018). Studies have shown that Aβ_1–42_, total tau protein, and threonine 181 phosphorylated tau protein (p-tau) (Asher and Priefer, 2022; Ossenkoppele et al., 2022) show very consistent changes in AD dementia and prodromal AD, and they have been included in the diagnostic criteria for AD studies and as evidence of the presence of AD pathology (Dubois et al., 2014).

Iron is essential for life processes and cell function. The main factors that affect iron levels in the brain with age include inflammation, vascular changes and metabolic changes. Iron accumulation has been observed in areas of the brain affected by AD, such as the parietal cortex, motor cortex and hippocampus (Ding et al., 2009; Bilgic et al., 2012; Luo et al., 2013; Langkammer et al., 2014; Tao et al., 2014; Ghadery et al., 2015; Masaldan et al., 2019b). According to histological observations, the intensity of iron accumulation in the frontal cortex is different in different types of AD. This can be used to distinguish sporadic (late onset) from familial (early onset) AD (Ghadery et al., 2015) and reflects the severity of the disease (van Duijn et al., 2017; Bulk et al., 2018).

Currently, researchers have developed different types of prognostic risk models for AD, including longitudinal measurement and event time-dependent prognostic risk models, as well as prognostic models based on baseline cognitive scores and MRI features (Li et al., 2018; Janelidze et al., 2020; Shu et al., 2021). Research indicates that there is also a model for AD prognosis by amyloid PET structure and shape that characterizes plasma P-tau181 (Caminiti et al., 2018; Janelidze et al., 2020). Some researchers have clearly proved the correlation between ferroptosis and AD pathogenesis (Bao et al., 2021; Jakaria et al., 2021; Yan et al., 2021; Ma et al., 2022). Thus, brain iron levels may underlie CSF ferritin signaling, providing further evidence that ferroptosis is crucial in AD (Diouf et al., 2019). However, the AD prognostic risk model based on the scoring formula of ferroptosis has not been reported yet.

Increasing age is associated with an increase in low-grade chronic inflammation, which contributes to the neurodegenerative process in AD (Onyango et al., 2021), and assessment of the extent of immune infiltration is a good indicator of the severity of inflammation. There is convincing evidence that neuroinflammation plays a central role in the pathogenesis of AD (Heppner et al., 2015; Ising et al., 2019; Sala Frigerio et al., 2019), which can aggravate Aβ and τ pathology (Ising et al., 2019). In this study, we downloaded the GSE138260 dataset through the Gene expression Omnibus (GEO) database and used the single sample Gene Set Enrichment Analysis (ssGSEA) algorithm to assess the immune infiltration of 28 immune cells in a sample of 36 cases. The prognostic risk model constructed in this study uses cutting-edge international statistical methods, and the information collected can help with clinical decision-making regarding the severity of AD.

## 2. Materials and methods

### 2.1. Data collection and preprocessing

Public gene expression datasets based on samples containing AD-related clinical diagnostic information were collected from the GEO database. We used brain tissue from 19 AD patients and 21 deceased healthy controls without any history of neurological or psychiatric disease (Nitsche et al., 2021). Due to the quality of the arrays, and did not send relevant data on the GEO platform. Four arrays (2 arrays in AD group and 2 arrays in control group) were excluded from further processing. We normalize the dataset using external data functions. The training set was normalized to GSE138260 after removing duplicate or unannotated outliers and probes.

### 2.2. ssGSEA algorithm evaluation sample

ssGSEA is an extension of GSEA method. Based on the bulk RNA gene expression profile, significant genes in 28 immune cell genes (Download reference gene sets for 28 types of immune cells)^1^ can be used as reference files by ssGSEA. Finally, the enrichment degree of 28 immune cells in 36 sample microenvironments in GSE138260 can be calculated by using R package “GSVA.” The enrichment fraction of 28 kinds of immune cells in 36 samples of GSE138260 was obtained by ssGSEA algorithm. We used “limma” package to analyze the difference in the enrichment fraction expression profiles of immune cells between the two groups. According to the threshold value of Fold Change (FC) > 1 and *p* < 0.05, cluster analysis was performed on 17 AD samples according to the up-regulated immune cells in the screening results.

### 2.3. Subtype differential gene screening

The infiltration of 28 immune cells were analyzed in AD samples and control samples, and Activated B cell and Type 17 helper cell were up-regulated. Cluster 1 group (early onset) and Cluster 2 group (late onset) in AD samples were divided dependent on enrichment levels of Activated B cell and Type 17 helper cell. The differential genes between Cluster 1 group and Cluster 2 group were determined according to FC > 1 and *p* < 0.05 by “limma” package. Overlap genes of disregulated genes between Cluster 1 vs. control and Cluster 2 vs. Cluster 1 by Venn diagram.

### 2.4. Enrichment analysis of modules

Gene Ontology (GO) enrichment and Kyoto Encyclopedia of Genes and Genome (KEGG) enrichment were performed on the differential genes selected at the initial stage of AD, with *p* value of 0.05 and adjusted *p* value of 0.05. GO enrichment and KEGG enrichment were performed on the differential genes selected during the AD development period, with *p* value of 0.05 and adjusted *p* value of 0.05.

### 2.5. Cell culture

Well-differentiated rat PC12 cells purchased from Wuhan Procell Life Science and Technology Co., Ltd. (Wuhan, China) and cultured with Dulbecco’s modified eagle’s medium (DMEM, Gibco BRL, United States) supplemented with 10% fetal bovine serum (FBS, Thermo Fisher, Australia) and antibiotics (100 U/mL penicillin G and 100 mg/mL of streptomycin) in a humidified incubator with a 5% CO_2_ air mixture at 37°C.

### 2.6. Cell proliferation assessment

Aβ_1–42_ was used to induce PC12 cells to construct a cellular model of AD. Aβ_1–42_ (MedChemExpress) was dissolved in Dimethylsulfoxide (DMSO) to prepare a 1 mM storage solution, which was stored frozen at −80°C. PC12 cells were isolated with 0.05% trypsin, centrifuged at 1500 rpm for 3 min, the supernatant was removed, and complete medium was added. Cells were inoculated into 96-well plates with 100 μL per well and incubated in a cell culture incubator. PC12 cells were then treated with different concentrations of Aβ_1–42_ (0, 20, and 40 μM) for 24 h. The morphology of cells treated with different concentrations of Aβ_1–42_ was observed under a microscope (Olympus, Tokyo, Japan). Cells were changed by adding 90 μL of double antibody-free medium (10% FBS, 90% DMEM) and 10 μL of Cell Counting Kit-8 (CCK-8) solution, (Abbkine, California, United States) and incubated for 3 h. Absorbance was measured at 450 nm using a 96-well plate (VICTOR Nivo; PerkinElmer, Finland).

### 2.7. Quantitative real-time polymerase chain reaction

Ribonucleic Acid (RNA)was extracted from cell lysates using the RNA Blood Mini Kit and RNeasy^®^ Mini Kit (QIAGEN) according to the manufacturer’s instructions. The RNA was reverse transcribed using the ReverTra Ace qPCR RT Kit and ReverTra Ace qPCR RT Master Mix with gDNA Remover (TOYOBO). The relative FC in expression of the target normalized to expression of the corresponding control was calculated by the comparative Ct method. Primers are described in Table 1.

**Table 1:** Primer sequences.

Name	Sequences
β-actin	F: 5′- GGGAAATCGTGCGTGACATT -3′ R: 5′- GGAACCGCTCATTGCCAAT -3′
POR	F: 5′- CTCCAAGACTACCCATCACTG -3′ R: 5′- GACTTCGCTTCGTACTCCAC -3′
RUFY3	F: 5′- AAGGGGATGGACAGATTACT -3′ R: 5′- TTTTGCCTGAAGGTTGTTT -3′

### 2.8. Web nomogram calculator construction and validation of the nine-hub-gene signature

Using R package “rms,” based on the expression data in GSE138260, a least absolute shrinkage and selection operator (LASSO) Cox regression analysis and Cox univariate analysis model is established. The corresponding network nomogram calculator based on ferroptosis AD prognostic risk model was constructed. The machine learning model calibration curve is constructed, and the AUC value of the receiver operating characteristic (ROC) curve is calculated with “pROC” package to verify the model.

### 2.9. Statistical analysis

Data were analyzed by GraphPad Prism 7.0 software and expressed as mean ± SD. Statistical comparisons between groups were performed using the least significant difference *t*-test or one-way ANOVA. The differences were considered significant at *p* ≤ 0.05.

## 3. Results

### 3.1. Schematic diagram of research flow

**Figure 1:**
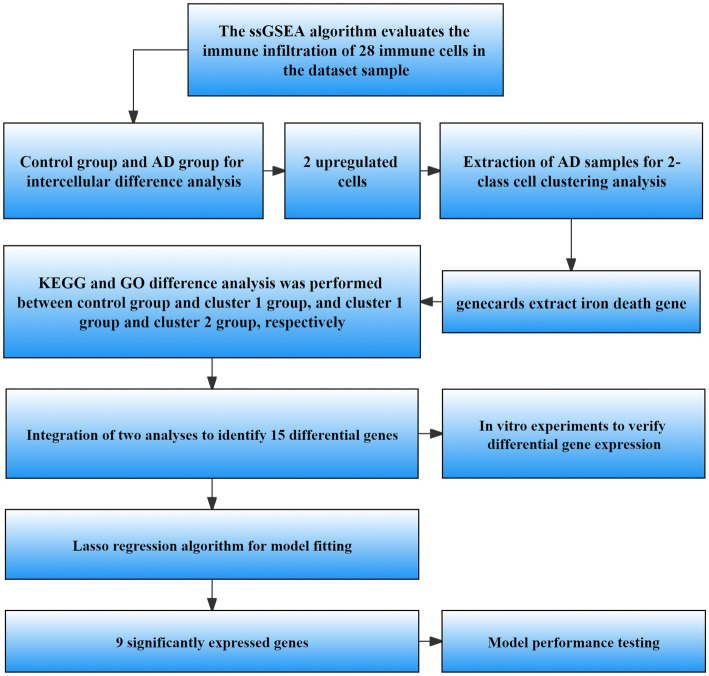
Flow chart of data processing and analysis. ssGSEA, Single sample gene set enrichment analysis; AD, Alzheimer’s disease; GO, Gene ontology; KEGG, Kyoto Encyclopedia of Genes and Genomes; LASSO, Least absolute shrinkage and selection operator.

Schematic representation of the workflow used in this study is shown in Figure 1.

### 3.2. Difference analysis results between AD group and control group

**Figure 2:**
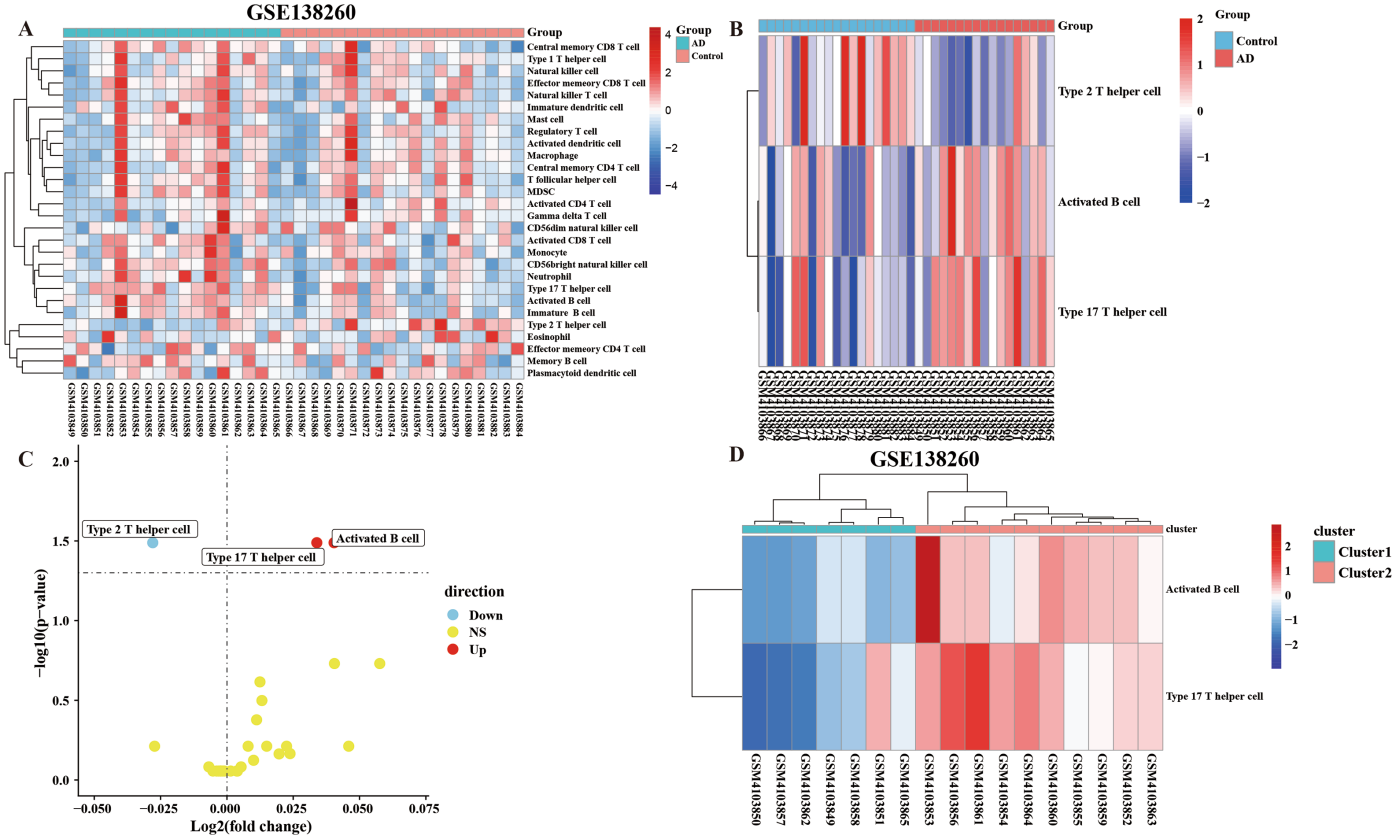
The ssGSEA algorithm was used to assess the immune infiltration of 28 immune cells in 36 samples. **(A)** The 28 kinds of immune cells were divided into the control group and the AD group for all heatmap analysis of the difference of cellular immune infiltration degree. **(B)** Immune heatmap for differential analysis of immune infiltration by dividing AD group and control group (FC > 1 and *p* < 0.05). **(C)** Volcano plot of differentially analyzed cells classified into two clusters by differential analysis of immune infiltration. **(D)** Heatmap of the cells screened by the differential analysis of immune infiltration in the AD group were classified into two clusters and subjected to subtype GO analysis.

In order to compare the difference between AD and control groups, we used the ssGSEA algorithm to perform the enrichment analysis of immune infiltration of immune cells. The results showed that there were differences between the cells of AD group and control group (Figure 2A). We defined *p* < 0.05, FC > 1 as cells with significant differences, and analysis by heat and volcano plots showed that the cells with significant differences were Type 2 T helper cells (down-regulated), Activated B cells and Type 17 T helper cells (up-regulated), respectively (Figures 2B,C). The up-regulated cells were divided into subtypes by enrichment analysis. It can be seen from the figure that there are differences in the degree of immune infiltration between the two clusters of cells after enrichment analysis (*p* < 0.05, FC > 1) (Figure 2D).

### 3.3. Results of difference analysis between control group and Cluster 1 group

**Figure 3:**
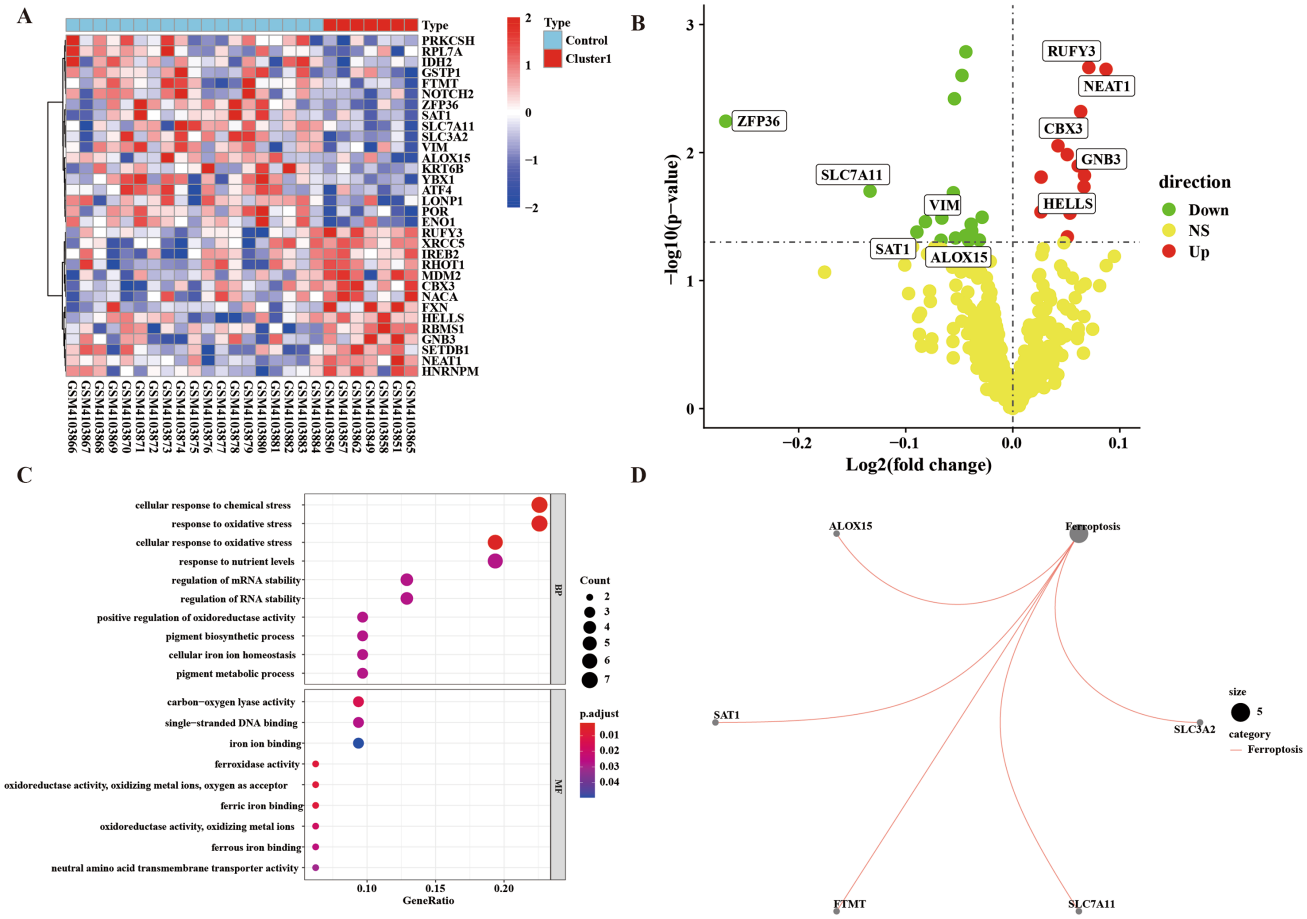
Gene difference enrichment analysis was performed between Cluster 1 group and control group. **(A)** Heatmap of differential gene enrichment analysis between Cluster 1 group and control group. **(B)** Volcanic map of difference analysis between Cluster 1 group and control group. **(C)** Bubble plot of GO analysis between normal group and Cluster 1 group. The size of each circle indicates the gene count. The color of circles represents different −log10 (values). **(D)** KEGG pathway diagram of enrichment analysis between normal group and Cluster 1 group.

We divided the up-regulated cells into two clusters, defined as the low-symptom group (Cluster 1 group) with a lower degree of immune infiltration, and defined as the high-symptom group (Cluster 2 group) with a higher degree of immune infiltration. We performed an enrichment analysis of genes in control group and Cluster 1 group, defined as significantly different according to *p* < 0.05, FC > 1. Analysis by heat map and volcano map showed that there were 14 up-regulated genes and 18 down-regulated genes (Supplementary material Table 1; Figures 3A,B). Meanwhile, KEGG and GO analyses were performed for up-regulated and down-regulated genes (Figures 3C,D). GO enrichment results showed that the differential genes of control group and Cluster 1 group were not only enriched in cellular to chemical stress, response to oxidative stress, and other biological processes. Additionally, it is enriched in carbon–oxygen lyase activity, single-stranded DNA binding, and other molecular functions. The results of KEGG showed that differential genes were enriched in the ferroptosis pathways.

### 3.4. Results of difference analysis between Cluster 1 group and Cluster 2 group

**Figure 4:**
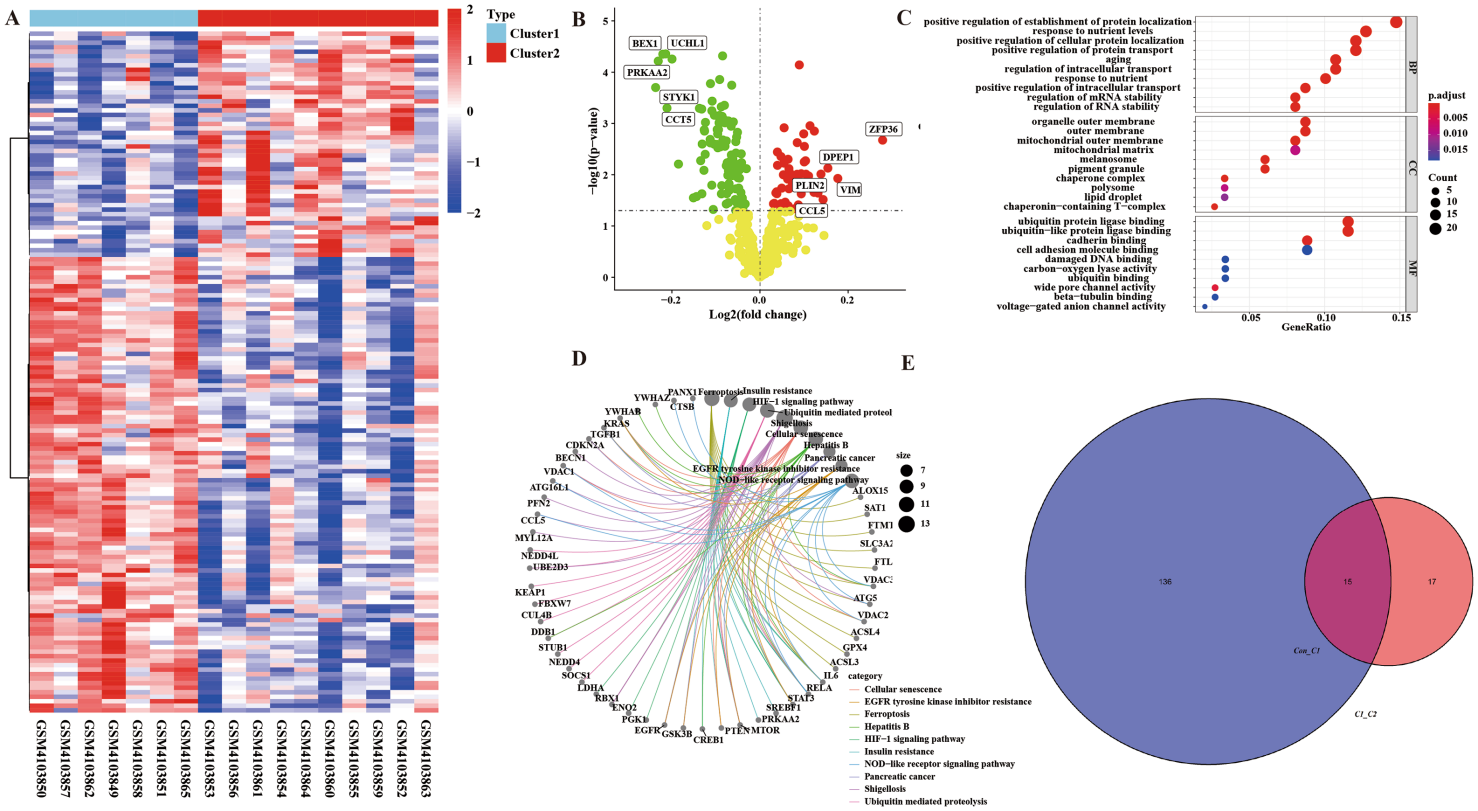
There were ferroptosis-related differentially regulated genes among the integrated control group, Cluster 1 group, and Cluster 2 group. **(A)** Heatmap of differential genes in Cluster 1 group and Cluster 2 group enrichment analysis. **(B)** Volcanic map of differential genes in Cluster 1 group and Cluster 2 group enrichment analysis. **(C)** Bubble map of GO analysis of differential genes between Cluster 1 and Cluster 2 groups. **(D)** KEGG pathway diagram of differential gene enrichment analysis between Cluster 1 group and Cluster 2 group. **(E)** Venn diagram of ferroptosis-related crossover genes between control group and Cluster 1 group, Cluster 1 group, and Cluster 2 group.

We performed enrichment analysis on Cluster 1 group and Cluster 2 group to compare differential genes. According to *p* < 0.05, FC > 1 was defined as having a significant difference. The analysis of heat map and volcano map showed that there were 50 up-regulated genes and 101 down-regulated genes (Supplementary material Table 2) (Figures 4A,B). At the same time, we performed KEGG analysis and GO analysis on up-regulated and down-regulated genes (Figures 4C,D). GO enrichment results showed that the differential genes of Cluster 1 group and Cluster 2 group were not only enriched in the positive regulation of the establishment of protein localization and other biological processes, it also enriched in the molecular functions of ubiquitin protein ligase binding and other components such as organelle outer membrane. The results of KEGG showed that differential genes were enriched in ferroptosis and insulin resistance pathways.

Through the intersection analysis of differential genes between the control group and Cluster1 group, and between the Cluster 1 group and Cluster 2 group, there are a total of 15 cross genes (Figure 4E and Table 2), and all these genes were associated with ferroptosis. We selected the representative genes (with the smallest *p* value and the largest FC value) for verification *in vitro* experiments. The representative gene among the down-regulated genes is Cytochrome p 450 reductase (POR), *p* = 0.001635428, FC = 0.970082793. The representative gene among the up-regulated genes is RUN and FYVE domain-containing protein 3 (RUFY3), *p* = 0.002168468, FC = 1.050454379.

**Table 2:** Integrate 15 expressed differentially regulated genes related to ferroptosis.

Gene	FC	Log2fc	*P* value	Direction
RUFY3	1.050454379	0.071013507	0.002168468	Up
HNRNPM	1.035896674	0.050880108	0.010377742	Up
SETDB1	1.043243996	0.061076618	0.012685491	Up
XRCC5	1.018515589	0.026468061	0.015563745	Up
IREB2	1.037747961	0.053456097	0.029743085	Up
RHOT1	1.035961013	0.050969710	0.045555780	Up
POR	0.970082793	−0.043820214	0.001635428	Down
SLC3A2	0.967640134	−0.047457486	0.002499460	Down
ZFP36	0.830397684	−0.268125674	0.005694192	Down
FTMT	0.955099867	−0.066276503	0.032483359	Down
VIM	0.945007628	−0.081602121	0.034651096	Down
SAT1	0.939810528	−0.089558166	0.041761802	Down
NOTCH2	0.969825064	−0.044203556	0.044567914	Down
KRT6B	0.963630571	−0.053447932	0.046521828	Down
ALOX15	0.954628885	−0.066988105	0.048446862	Down

### 3.5. Aβ_1–42_ interferes with PC12 cells to construct the model of Alzheimer’s disease

**Figure 5:**
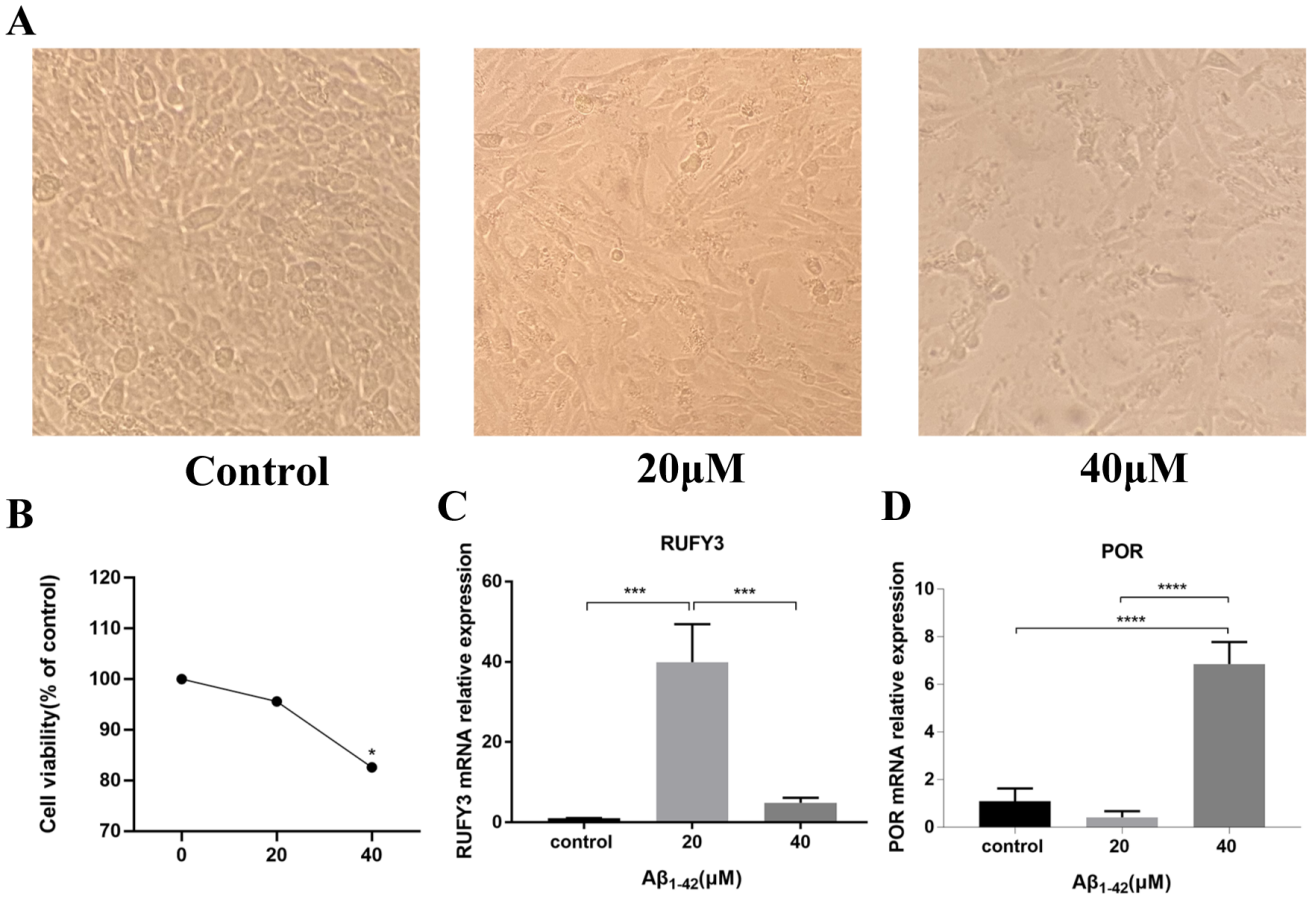
Aβ_1–42_ interfered with PC-12 cells to verify the expression of representative genes selected by differential analysis. **(A)** The differences among the three groups of cells were observed under the electron microscope. **(B)** CCK-8 experiment verified the survival of cells under the condition of Aβ_1–42_ in three concentration groups of 0, 20, and 40 μM. **(C)** Expression of the representative up-regulated gene RUFY3 under the condition of Aβ_1–42_ in three concentration groups of 0, 20, and 40 μM. **(D)** Expression of the representative down-regulated gene POR under the condition of Aβ_1–42_ in three concentration groups of 0, 20, and 40 μM. *p < 0.05, ***p < 0.001, ****p < 0.0001.

In order to verify the authenticity of our data analysis, we established an AD model by intervening PC12 cells with Aβ_1–42_ to verify the expression of representative genes in the up-regulated and down-regulated genes in the model. The results of CCK-8 experimental study showed that the survival rate of PC12 cells gradually decreased under the intervention conditions of Aβ_1–42_ in three different concentration groups of 0, 20, and 40 μM (Figure 5A). The cell survival rate was significantly decreased in the 40 μM group compared with the 0 μM group, and the difference was statistically significant (*p* < 0.05) (Figure 5B).

The results of Real Time Quantitative PCR (RT-qPCR) experiments showed that RUFY3, a representative gene among the up-regulated genes, exhibited an ascending trend followed by a descending trend (Figure 5C). The expression of RUFY3 in the 20 μM group was significantly increased compared with the 0 μM group, and the difference was statistically significant (*p* < 0.0001). Compared with the 20 μM group, the expression of RUFY3 in the 40 μM group was significantly reduced, and the difference was statistically significant (*p* < 0.0001); the expression of RUFY3 also increased in the 40 μM group compared with the 0 μM group. POR, a representative gene among the down-regulated genes, showed a trend of decreasing first and then increasing (Figure 5D). Compared with the 0 μM group, the expression of POR in the 20 μM group tended to decrease; compared with the 20 μM group, the expressions of the other groups were significantly higher (*p* < 0.0001).

### 3.6. Construction and validation of the prognostic risk model

**Figure 6:**
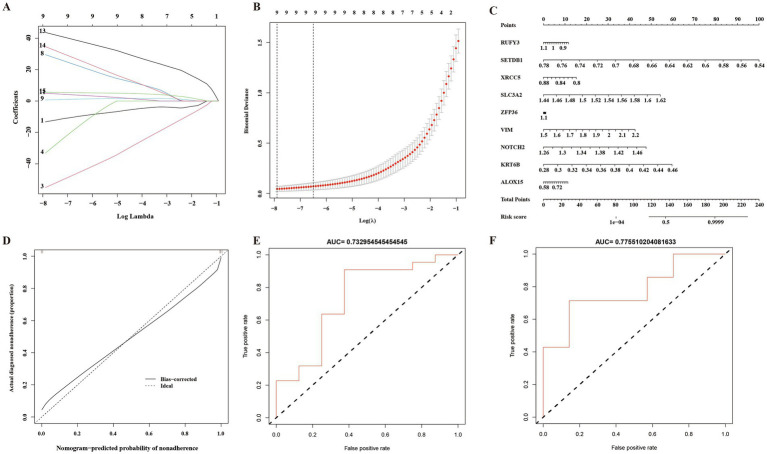
A prognostic model of Alzheimer’s disease based on ferroptosis. **(A)** 10-fold cross-validation for tuning parameter selection in a LASSO Cox regression model. **(B)** Coefficient distribution trend of LASSO Cox regression. **(C)** Density plots of total points and representative genes show their distribution. **(D)** The importance of representative gene features based on random forest algorithm and the ideal number of gene features. **(E)** Control group and Cluster 1 group ROC curve analysis of prognostic risk model. AUC: Area under the time-dependent receiver operating characteristic curve. **(F)** Cluster 1 group and Cluster 2 group ROC curve analysis of prognostic risk model. AUC: Area under the time-dependent receiver operating characteristic curve.

In this study, LASSO regression analysis was used for feature selection. The expression data of 15 hub genes were fed into the LASSO regression model, and 10-fold cross-validation was performed to detect the best classification accuracy (Figures 6A,B). Therefore, 9 hub gene (RUFY3, SETDB1, XRCC5, SLC3A2, ZFP36, VIM, NOTCH2, KRT6B, ALOX15) characteristics were obtained based on LASSO regression analysis for further analysis. We performed weight analysis on representative genes (Table 3). A nomogram for prognosticating the prognosis of AD patients was constructed using 9 hub genes (Figure 6C). Density plots of total points and representative genes show their distribution. The importance of each variable is ranked according to the standard deviation on the nomogram scale. A scoring formula was used to calculate the weight of each gene. The formula for calculating the sum of the weight values of each gene using the scoring formula is as follows: RiskScore = (−13.2689334*Gene 1) + (−55.1575122*Gene 3) +(−33.2441371*Gene 4) + (29.5543714*Gene 8) + (0.6545401*Gene 9) + (4.9532565*Gene 11) + (43.6929789*Gene 13) + (34.6422127*Gene 14) + (5.5404864*Gene 15) + (−50). There is a good agreement between the calibration curve and the actual scale (Figure 6D). Finally, we used the GSE28146 dataset for external data validation of the model. The ROC curve results showed that the risk score of the prognostic risk model had good predictive ability. The ROC results of control group and Cluster 1 group showed: AUC = 0.732954545454545 (Figure 6E). The ROC results of Cluster 1 group and Cluster 2 group showed: AUC = 0.77551020408163 (Figure 6F).

**Table 3:** List of weight genes.

Number	Gene	(Intercept) −50.1600862
1	RUFY3	−13.2689334
3	SETDB1	−55.1575122
4	XRCC5	−33.2441371
8	SLC3A2	29.5543714
9	ZFP36	0.65454010
11	VIM	4.95325650
13	NOTCH2	43.6929789
14	KRT6B	34.6422127
15	ALOX15	5.54048640

## 4. Discussion

Currently, many researchers have developed different AD prognostic risk models, including AD prognostic risk model constructed by the psychological theory of amnestic mild cognitive impairment, integrated radiomics model, AD prognostic risk model constructed by combining tau-PET and fMRI, etc. (Ben Bouallegue et al., 2017; Dimitriadis et al., 2018; Zhang et al., 2018; Yi et al., 2020; Shu et al., 2021; Biel et al., 2022). In these AD prognostic risk models currently established, most of the research data are screened through databases such as PubMed, Embase, ADNI, ADNI-2, and Web of Science. However, GEO databases are rarely used. In recent years, some researchers have used the GEO public database to screen the marker genes related to AD. However, no further studies have been conducted to establish a prognostic risk model for AD. Therefore, in this study, we screened the genes associated with AD through the data of GEO database and constructed the prognostic model of AD through the screened genes.

In this study, we conduct analysis through the GSE138260 dataset in the GEO database. The results of this study showed that 32 genes with differential immune infiltration were screened out from the control group and Cluster 1 group, and KEGG enrichment analysis results found 32 genes related to ferroptosis in the KEGG pathway. This indicated that the control group and AD Cluster 1 group had obvious different genes, which was clinically manifested as whether or not they had AD. Cluster 1 group and Cluster 2 group were analyzed for differences, and 151 differential genes were screened out. KEGG enrichment analysis results found that these 151 genes were related to the KEGG pathway of ferroptosis. This shows that there are obvious differential genes between Cluster 1 group and Cluster 2 group in the AD group, and clinically, the clinical symptoms of Cluster 2 group are more severe than those of Cluster 1 group. Zhang et al. (2021) used the GSE5281 microarray dataset from the GEO database and screened the hub genes by logistic regression and LASSO analysis. Zhao et al. (2022) also used the GEO database and finally identified six genes as new biomarkers for Alzheimer’s disease through a comprehensive analysis of weighted gene co-expression network analysis. Our research methods are similar to previous screening methods, and the above research methods have proved the accuracy and feasibility of these screening methods.

In our study, we analyzed the data set GSE138260 in the GEO database. In the GSE138260 dataset, we divided the samples into control group and AD group. We used ssGSEA algorithm to analyze the difference of immune infiltration. The results showed that the control group and the AD group had differences in the degree of immune infiltration. The analysis revealed significant changes in immune cells in AD, including Type 2T helper cells, Activated B cells, and Type 17T helper cells. At the same time, we grouped the AD group again into Cluster 1 group and Cluster 2 group. The results of this study showed that 32 immune infiltrating differential genes were screened out between the control group and the Cluster 1 group, which may cause cell damage through influencing the ferroptosis pathways and ultimately lead to the occurrence of AD. Cluster 1 group and Cluster 2 group were analyzed, and 151 differential genes were screened. This indicates that there are obvious differential genes between Cluster 1 and Cluster 2 in the AD group, which are manifested in that the differential genes are enriched in the ferroptosis pathways and have the molecular function of protein ubiquitination, which may be the inducement of the deterioration of AD. These data reveal the important role of infiltration of specific immune cell types in AD and provide guidance for the pathogenesis and subtype construction of AD. Therefore, in this study, our methods of gene screening through GEO database are accurate and feasible.

Current studies have shown that iron is a promoter of neurodegeneration related to β-amyloid pathology, and iron may play a role in promoting the development and progression of AD in the prodrome phase. Elevated brain iron levels are associated with the pathology of AD, cognitive decline, and possibly through an iron-mediated programmed cell death mechanism, ferroptosis, leading to neuronal loss (Masaldan et al., 2019a). A growing number of studies have shown that ferroptosis is associated with cancer and neurodegenerative diseases such as glioblastoma, Alzheimer’s disease, Parkinson’s disease, and stroke (Wang Y. et al., 2022). Ferroptosis is mainly regulated through iron homeostasis, glutathione metabolism, and lipid peroxidation. Therefore, in this study, we identified 15 model-significant genes using Venn diagram by adjusting the expression profile of ferroptosis genes. At the same time, we used *in vitro* experiments to verify the screened significant genes to ensure the accuracy of the genes we selected. GO and KEGG pathway analyses showed that these DEGs were located in the signaling pathways related to ferroptosis, all of which were consistent with previous findings.

In the current studies on AD model building, few studies have validated the screened genes through *in vitro* experiments, mostly through another dataset or GC patients (Chen W. et al., 2021; Liu et al., 2021; Chen et al., 2022). Chen W. et al. (2021) successfully constructed an AD prediction model using the ADNI database and combining clinical and imaging histological features, however, it was not validated by *in vitro* experiments. Most of the current prognostic risk models using *in vitro* experiments for validation are mainly tumor prognostic risk models, and there are very few studies using *in vitro* experiments for validation in prognostic risk models of AD (Barbie et al., 2009; Xiang et al., 2019). Therefore, the use of *in vitro* experiments can more effectively test the accuracy of our gene expression trend screening database. In this study, we used CCK-8 and RT-qPCR to validate the genes we screened. The results of CCK-8 showed that the survival rate of PC12 cells decreased with the increase of Aβ_1–42_ concentration, which indicated that Aβ_1–42_ successfully constructed the model of AD. RT-qPCR results showed that RUFY3, a representative gene among the up-regulated genes, exhibited an upward and then downward trend, and a representative gene among the down-regulated genes, the expression of POR decreased first and then increased.

RUFY3 is a new member of actin-related proteins specifically expressed in mouse neurons and is important for neural axon morphogenesis. Studies have shown that RUFY3 is expressed only in neurons of mouse brain tissue and not in NPCs or glial cells, suggesting that it has a unique role in neuronal development. In addition, we found that RUFY3 interacts with Fascin and Drebrin and co-distributes with F-actin in axonal growth cones. These findings have important implications for understanding how neuronal axon formation and growth cone morphogenesis are controlled at the molecular level (Wei et al., 2014).

POR is the representative gene among down-regulated genes, which exhibits an ascending and then descending trend. Studies have mentioned POR, a 678-amino acid microsomal flavoprotein, is an obligate redox partner for all microsomal P450 cytochromes (Laursen et al., 2011; Borkowski et al., 2021). It has been shown that patients with Alzheimer’s disease have elevated levels of components of the cytochrome P450/soluble epoxide hydrolase pathway (Borkowski et al., 2021). Immunoreactive bands corresponding to cytochrome NADPH P450 reductase are significantly increased after exposure of neuroblastoma cells to amyloid peptides (Pappolla et al., 2001). Our study shows that POR increases abruptly during the initial AD phase and will tend to decrease as the disease becomes more severe in AD patients. This also opens up the possibility of POR as a potential biomarker for the prediction of the stage of AD development. The results of the *in vitro* experiments were consistent with the expression results of the genes we screened out, indicating that the screening methods we used in this study and the genes we screened out were correct.

There is almost no precedent in the world for constructing a prognostic risk model by calculating the gene weights using the scoring formula of the AD prognostic risk model. At present, only some researchers have mentioned the construction of related models in tumor-related research. Therefore, we propose a scoring formula for the AD prognostic risk model and present the weights of the genes calculated by the scoring formula in the form of a column line graph. The severity of disease in AD patients can be assessed by measuring the level of gene expression and calculating it using a scoring formula.

In the existing studies, LASSO regression is mostly used as a variable screening method to obtain explanatory variables with non-zero coefficients by LASSO regression, and then multiple regressions are performed to build prediction models using these screened variables, which is actually a special case of RELAXED LASSO (*γ* = 0). LASSO regression models can help avoid overfitting in large data sets where the number of variables far exceeds the number of samples (Wang Q. et al., 2022). This method can make up for the deficiency of the least square estimation method and the local optimal estimation of stepwise regression, and effectively solve the problem of multicollinearity among the features (Dutta et al., 2020). In order to optimize our model more comprehensively and systematically, we have also tried to consider elastic net. Elastic net can combine L1 and L2 penalties and avoid some of the instability issues seen on Lasso for correlated predictors (Li et al., 2021, 2022; Xie et al., 2023). In addition, we used the GSE28146 dataset for external data validation of the model. However, according to our test, the accuracy of elastic net is relatively poor compared with LASSO regression model. The ROC curve results showed that the risk score of the prognostic risk model by elastic net had poor predictive ability. Therefore, in this study, we directly use the parameters of LASSO regression to model predictions. Our results show that 9 of the 15 hub genes have the best model fit for AD and can be used for model construction. Meanwhile, we used the importance of representative gene features based on the random forest algorithm and the ideal amount of gene feature analysis, and the results showed that the calibration curve overlaps relatively well with the straight line of y = x, which indicates that the calibration degree of the model we constructed in this study is very good. The ROC curve analysis showed that this prognostic column line plot had good classification ability, which fully assessed the goodness of fit of our model.

The AD prognostic risk model we constructed has the following innovations: The method of subgroup analysis improves the feasibility of AD prognostic risk mode. Bioinformatics combined with molecular biological evaluation increases the reliability of the model. The construction of the model makes it possible to conduct targeted intervention on AD quickly and simply. Most importantly, this study greatly saves the economic cost of clinical prognostic diagnosis of AD patients, which is undoubtedly very informative. Compared to previous studies, our findings support an urgent need to revise current diagnostic criteria for patient outcomes. At the early stage of the prognostic strategy, gene sequencing is preferentially initiated to target AD therapy.

However, our model also has the following limitations: we constructed our model based on retrospective data, and prospective clinical validation is needed in the future; although a scoring model for specific formulas was constructed, further research is needed on the pathogenesis of ferroptosis in AD; In addition, LASSO also has disadvantages. When there is a set of highly correlated features, Lasso regression method tends to select one feature while ignoring all other features, which will lead to the instability of the results (Dai et al., 2021). In the future, we may consider further optimizing the model to enhance its stability and improve its accuracy.

We will further expand the number of our datasets, conduct repeated verifications, and classify the severity of AD in more detail. The accuracy and sensitivity of the model will be further improved. At the same time, it pays more attention to the research of mechanism, and jointly evaluates our model from multiple dimensions such as population, animals, and cells. Through target genes, explore the interaction among ferroptosis genes, determine the regulatory relationship of each gene, and screen out more accurate ferroptosis pathways that play a role in AD. At the same time, our model should be combined with other investigators’ prognostic risk models of clinical biomarkers such as cerebrospinal fluid proteins and amyloid, and prognostic models of scale assessment categories for a multidimensional, multi-method joint assessment of AD prognosis to further improve the accuracy and usefulness of the model.

## 5. Conclusion

In this study, we successfully constructed a ferroptosis-based prognostic model for Alzheimer’s disease (AD). Although the range of ferroptosis genes is wide, our model identified the most representative 9 targeted genes, which further clarified the specific genes that play a role in the ferroptosis pathways. The hub genes allow for faster and easier evaluation of disease prognosis and provide the possibility of targeted interventions in these genes, which can aid in the treatment of AD. Additionally, our study focused more on gene screening, as compared to previous studies which mainly interpreted the role of ferroptosis in the pathogenesis of AD through proteins or their related pathways (Chen K. et al., 2021; Greenough et al., 2022; Ma et al., 2022). The genes we identified are involved earlier in the disease process than protein production and subsequent pathogenic mechanisms, allowing for earlier screening of AD progression. Therefore, the emergence of gene scoring models represents a new direction for future research on AD development.

The establishment of this research model can help clinicians make decisions on the severity of AD. Clinicians can tailor follow-up strategies or treatment regimens based on patients’ predicted risk of recurrence to improve long-term outcomes. Moreover, it provides guidance for medical institutions to effectively allocate and control medical expenses.

## Data availability statement

The datasets presented in this study can be found in online repositories. The names of the repository/repositories and accession number(s) can be found in the article/Supplementary material.

## Author contributions

X-LW, S-YW, and Y-HW conceived the idea of the study. R-QZ and Z-ML conducted the data analysis and drafted and revised the article. H-QL, Y-TL, F-FZ, and X-XH participated in the experiments and revised the article. All authors contributed to the article and approved the submitted version.

## Conflict of interest

The authors declare that the research was conducted in the absence of any commercial or financial relationships that could be construed as a potential conflict of interest.

## Publisher’s note

All claims expressed in this article are solely those of the authors and do not necessarily represent those of their affiliated organizations, or those of the publisher, the editors and the reviewers. Any product that may be evaluated in this article, or claim that may be made by its manufacturer, is not guaranteed or endorsed by the publisher.

## Footnotes

^1^ http://cis.hku.hk/TISIDB/data/download/CellReports.txt
