# Supplementary figures and images for: Constructing a prognostic risk model for Alzheimer’s disease based on ferroptosis
Source: Front Aging Neurosci. 2023 Apr 27;15:1168840. doi: 10.3389/fnagi.2023.1168840 (PMC10172508; doi:10.3389/fnagi.2023.1168840)

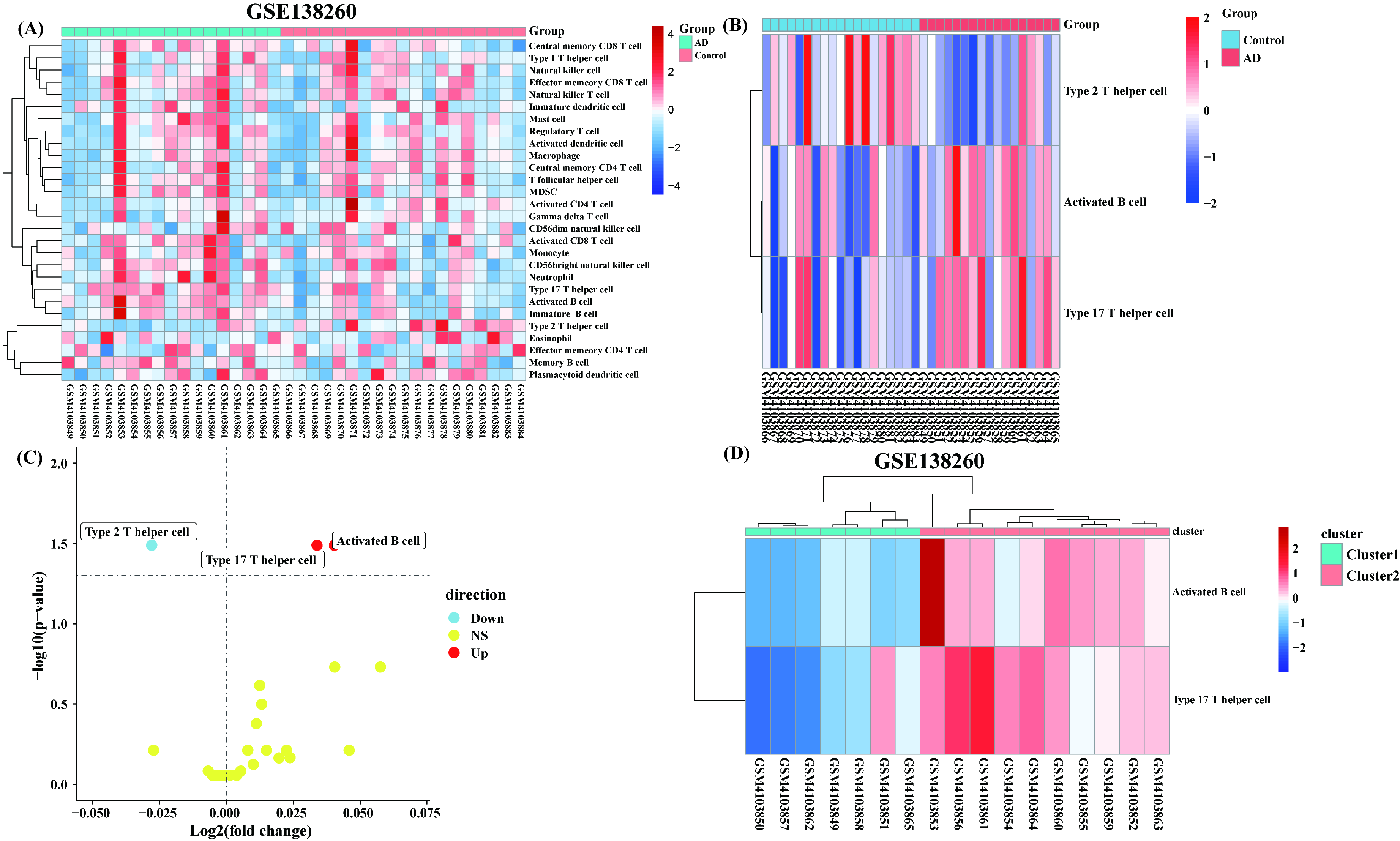

Supplement: Supplementary file 1 [file Presentation_1.zip › Supplementary Material Presentation/Figure 2.tif]

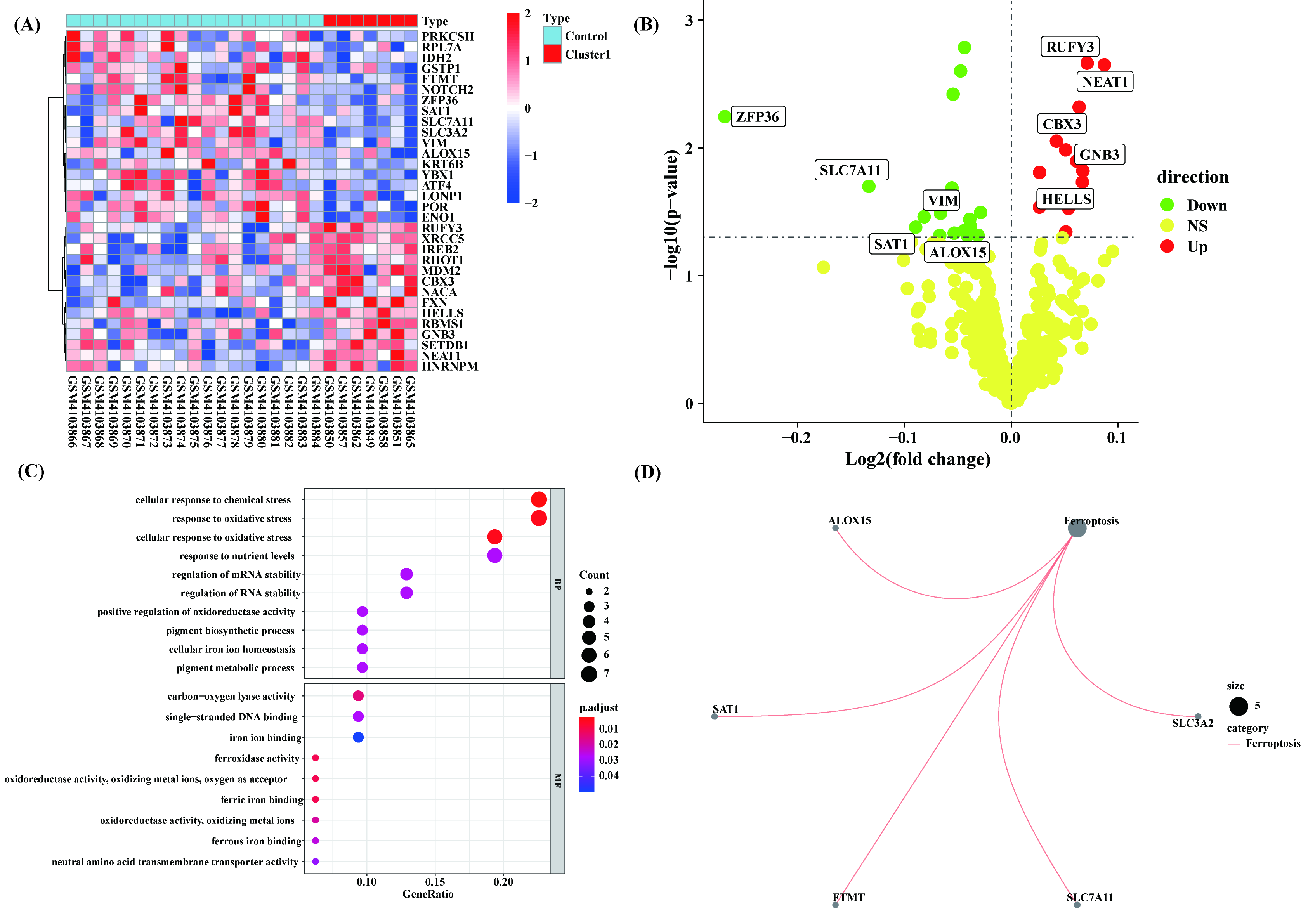

Supplement: Supplementary file 1 [file Presentation_1.zip › Supplementary Material Presentation/Figure 3.tif]

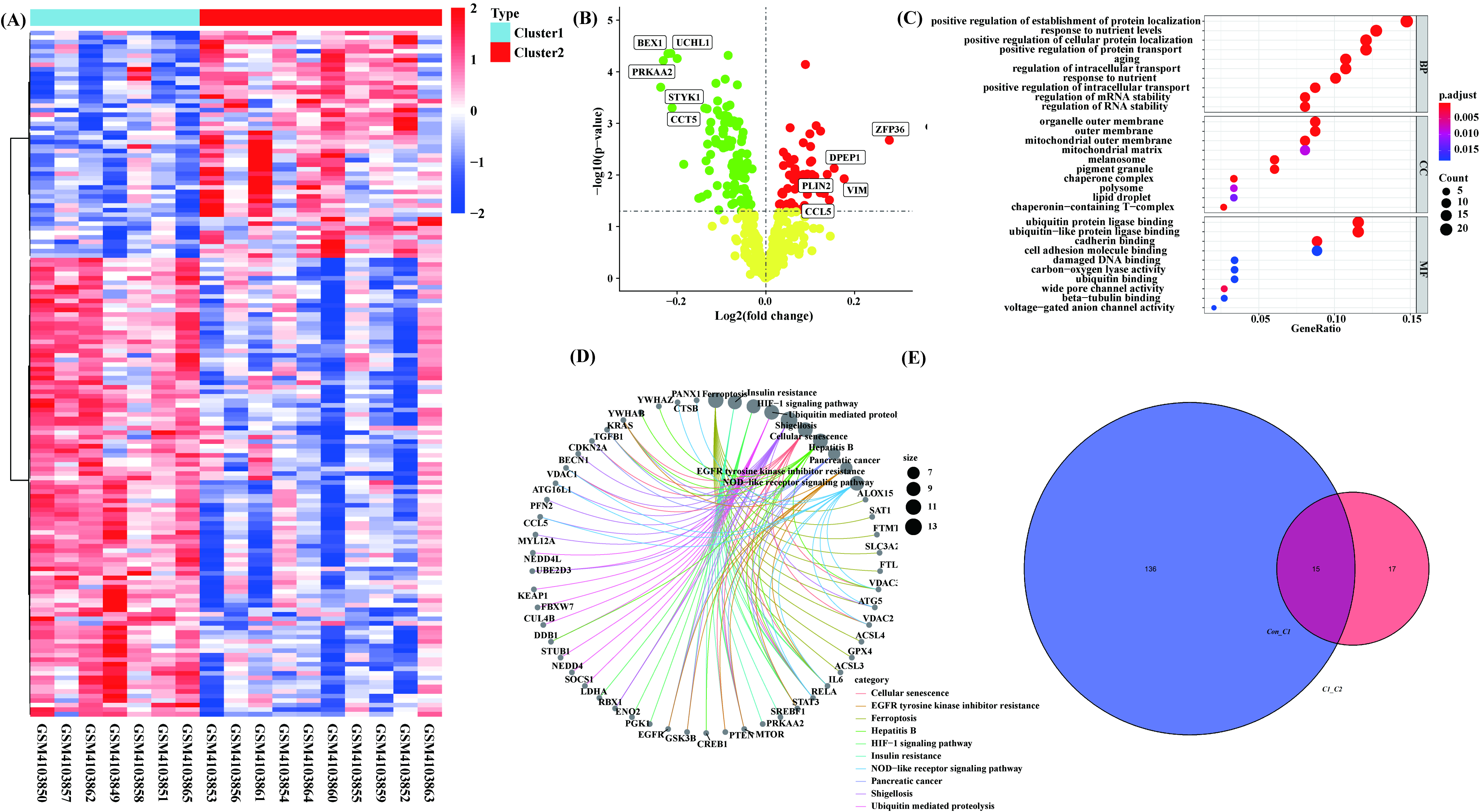

Supplement: Supplementary file 1 [file Presentation_1.zip › Supplementary Material Presentation/Figure 4.tif]

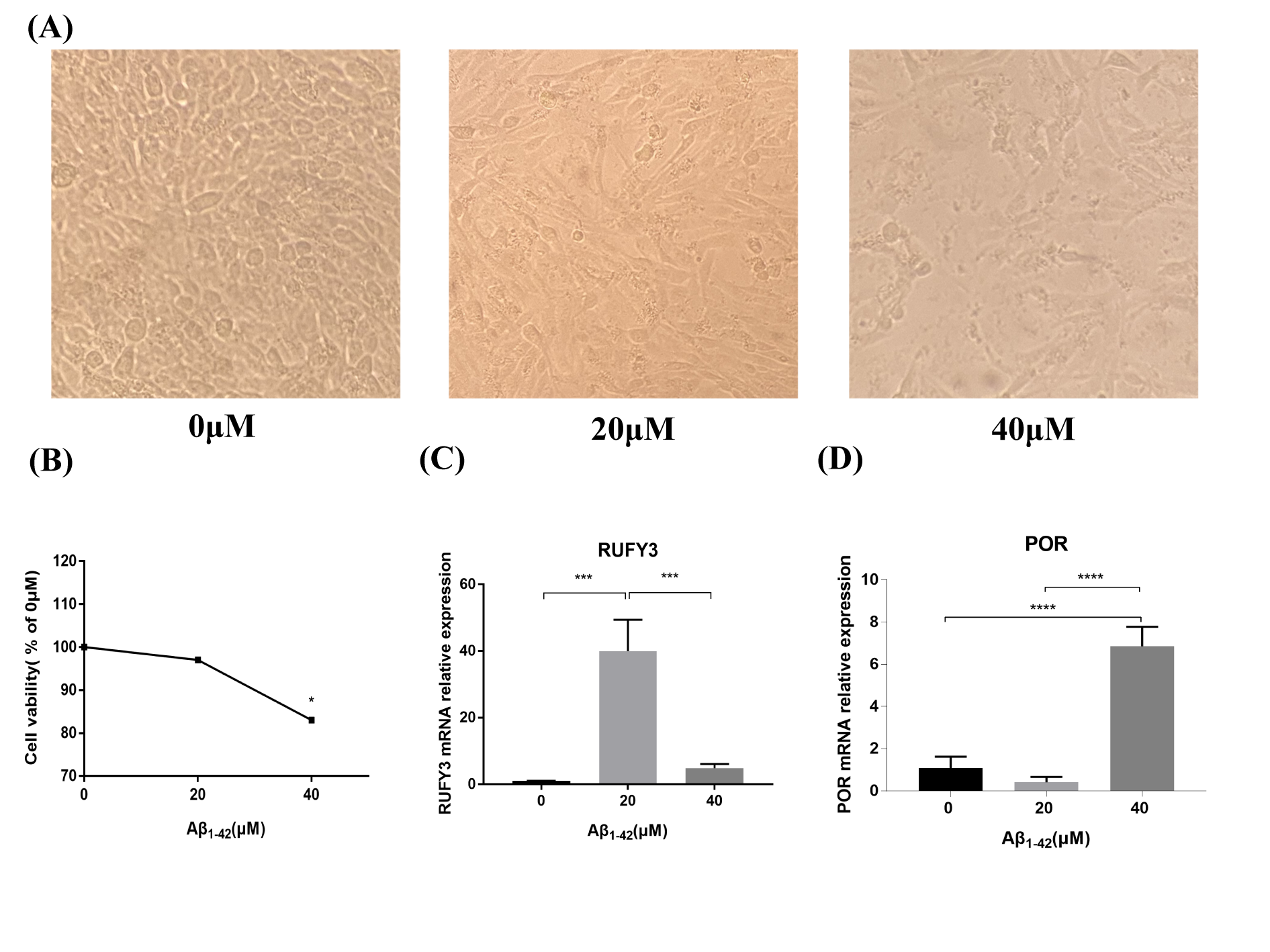

Supplement: Supplementary file 1 [file Presentation_1.zip › Supplementary Material Presentation/Figure 5.tif]

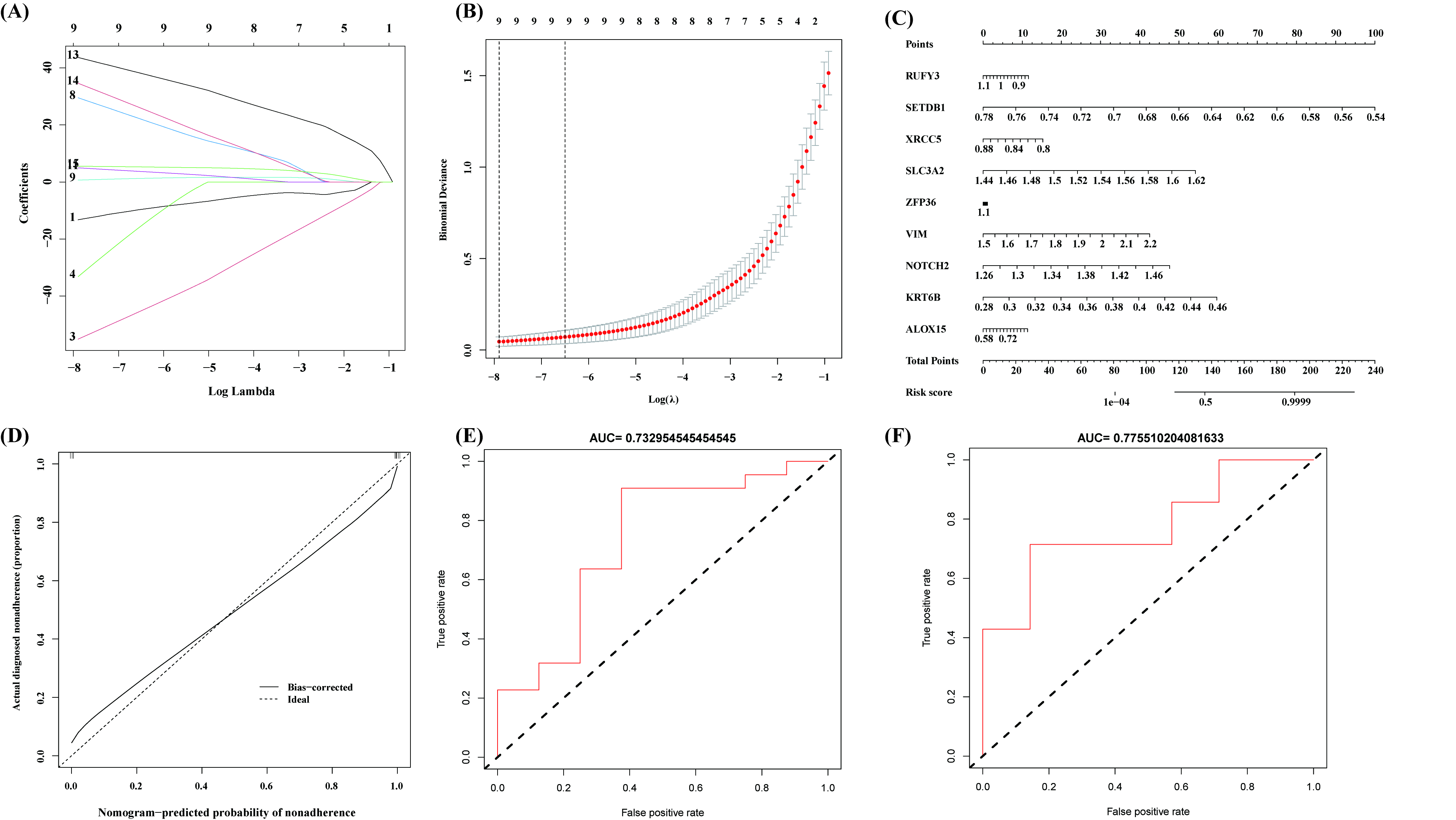

Supplement: Supplementary file 1 [file Presentation_1.zip › Supplementary Material Presentation/Figure 6.tif]
