# Supplementary material for: Constructing a prognostic risk model for Alzheimer’s disease based on ferroptosis
Source: Front Aging Neurosci. 2023 Apr 27;15:1168840. doi: 10.3389/fnagi.2023.1168840 (PMC10172508; doi:10.3389/fnagi.2023.1168840)
Supplement: Supplementary file 2 [file Presentation_2.pdf]

Supplementary Material

Constructing a prognostic risk model for Alzheimer's disease based on ferroptosis

Xiao-Li Wang [1†], Rui-Qing Zhai [2†], Zhi-Ming Li [1], Hong-Qiu Li [1], Ya-Ting Lei [1], Fang-Fang Zhao [1], Xiao-Xiao Hao [1] , Sheng-Yuan Wang [1*], Yong-Hui Wu [1*]

*** Correspondence:** Sheng-Yuan Wang: wangshengyuan@163.com

1 Supplementary Figures and Tables

Supplementary Table 1. Enrichment analysis of differential genes between control and Cluster 1 groups.

Gene	FC	Log2fc	*P* value	*P* adj	Direction
RBMS1	1.043456007	0.061369777	0.001611087	0.164131197	Up
FXN	1.03381734	0.047981305	0.00167177	0.164131197	Up
RUFY3	1.050454379	0.071013507	0.002168468	0.164131197	Up
NEAT1	1.062216684	0.087078095	0.00223952	0.164131197	Up
CBX3	1.044961195	0.063449368	0.004790212	0.235917923	Up
MDM2	1.029705659	0.042232003	0.008858781	0.349035981	Up
HNRNPM	1.035896674	0.050880108	0.010377742	0.371711838	Up
SETDB1	1.043243996	0.061076618	0.012685491	0.416506966	Up
GNB3	1.047517168	0.066973889	0.015114477	0.438008242	Up
XRCC5	1.018515589	0.026468061	0.015563745	0.438008242	Up
HELLS	1.047187496	0.066519776	0.018531965	0.477793436	Up

Gene	FC	Log2fc	*P* value	*P* adj	Direction
NACA	1.018415018	0.0263256	0.029088443	0.596933641	Up
IREB2	1.037747961	0.053456097	0.029743085	0.596933641	Up
RHOT1	1.035961013	0.05096971	0.04555578	0.596933641	Up
POR	0.970082793	-0.043820214	0.001635428	0.164131197	Down
SLC3A2	0.967640134	-0.047457486	0.00249946	0.164131197	Down
ENO1	0.962926472	-0.054502455	0.003802506	0.214026749	Down
ZFP36	0.830397684	-0.268125674	0.005694192	0.249279092	Down
SLC7A11	0.911721139	-0.133335469	0.020017173	0.477793436	Down
YBX1	0.962242208	-0.055528011	0.020615453	0.477793436	Down
LONP1	0.980339072	-0.028647271	0.032045098	0.596933641	Down
FTMT	0.955099867	-0.066276503	0.032483359	0.596933641	Down
VIM	0.945007628	-0.081602121	0.034651096	0.596933641	Down
PRKCSH	0.973385964	-0.038916124	0.036288607	0.596933641	Down
IDH2	0.973755689	-0.038368243	0.040955669	0.596933641	Down
SAT1	0.939810528	-0.089558166	0.041761802	0.596933641	Down
NOTCH2	0.969825064	-0.044203556	0.044567914	0.596933641	Down
RPL7A	0.973088047	-0.039357745	0.045043823	0.596933641	Down
KRT6B	0.963630571	-0.053447932	0.046521828	0.596933641	Down
GSTP1	0.978573934	-0.03124724	0.048253988	0.596933641	Down

Gene	FC	Log2fc	*P* value	*P* adj	Direction
ALOX15	0.954628885	-0.066988105	0.048446862	0.596933641	Down
ATF4	0.971847413	-0.041198276	0.048664069	0.596933641	Down

Supplementary Table 2. Enrichment analysis of differential genes between Cluster 1 and Cluster 2 groups.

Gene	FC	Log2fc	*P* value	*P* adj	Direction
NOTCH2	1.063820766	0.089255105	7.22E-05	0.00474285	Up
RTL1	1.08184332	0.113491573	0.001109032	0.013944241	Up
RPL38	1.038686194	0.054759855	0.001215203	0.014508784	Up
YAP1	1.089274233	0.12336721	0.001409277	0.014611975	Up
ALOX15	1.071203179	0.099232147	0.00159545	0.015331883	Up
ZFP36	1.212797759	0.278338993	0.002115095	0.018518828	Up
ALOX15B	1.060320746	0.084500744	0.002368342	0.019242937	Up
HSPB1	1.072385875	0.100824122	0.002801678	0.020441874	Up
RPS13	1.027780468	0.039532141	0.003607637	0.024936998	Up
CTSB	1.033600141	0.047678172	0.004426801	0.028131608	Up
EGFR	1.045126285	0.063677277	0.004994516	0.03098406	Up
SAT1	1.074292181	0.103386424	0.005347833	0.03158066	Up
FTMT	1.072111136	0.100454464	0.005628683	0.032613251	Up

Gene	FC	Log2fc	*P* value	*P* adj	Direction
MUC1	1.038338668	0.054277074	0.005933867	0.032928785	Up
POR	1.027874171	0.039663665	0.006558352	0.03415185	Up
DPEP1	1.112684089	0.154044044	0.007425452	0.037033266	Up
PDCD1	1.046575758	0.065676747	0.007603788	0.037065416	Up
CYGB	1.047564674	0.067039314	0.00898483	0.041163058	Up
DDR2	1.066987669	0.093543504	0.009549751	0.042822977	Up
PLIN2	1.100676613	0.138390657	0.009721848	0.042822977	Up
PSME1	1.074930796	0.104243783	0.009760431	0.042822977	Up
SOX2	1.060179606	0.084308694	0.009781898	0.042822977	Up
KRT6B	1.037471543	0.053071766	0.010023933	0.043400324	Up
AKR1C3	1.0794602	0.110310052	0.01057625	0.044330238	Up
SLC3A2	1.045112286	0.063657952	0.010997373	0.045184989	Up
SOCS1	1.044705761	0.063096667	0.011009541	0.045184989	Up
WWTR1	1.058432822	0.081929705	0.011140912	0.045252778	Up
VIM	1.130217561	0.17660051	0.011809279	0.04652856	Up
ETV4	1.044499684	0.062812056	0.012155898	0.047420038	Up
CDKN2A	1.052205884	0.073417023	0.014516678	0.056074229	Up
LGALS3	1.059383788	0.083225337	0.016127522	0.060516607	Up
IL6	1.075052167	0.104406668	0.016845015	0.062027439	Up

Gene	FC	Log2fc	*P* value	*P* adj	Direction
RELA	1.034831217	0.049395481	0.018256942	0.065992982	Up
STAT3	1.046637729	0.06576217	0.01879566	0.066940504	Up
ACTBL2	1.026581538	0.03784822	0.021523474	0.074388146	Up
TGFB1	1.088781204	0.122714066	0.022082388	0.075602267	Up
HMGN1	1.025282216	0.036021076	0.022635121	0.075602267	Up
STOM	1.095569101	0.131680483	0.022805535	0.075602267	Up
ADCY10	1.026918486	0.038321669	0.023428142	0.075602267	Up
MYL12A	1.067075497	0.093662252	0.023583531	0.075602267	Up
CCL5	1.104389382	0.143248923	0.030494222	0.093137391	Up
NEDD4	1.040484606	0.05725562	0.034298826	0.10145547	Up
COPZ1	1.022657785	0.032323453	0.03612632	0.102401225	Up
SREBF1	1.033744008	0.047878967	0.036604355	0.102578616	Up
LPIN1	1.021409234	0.030561007	0.03693586	0.102578616	Up
FTL	1.062208799	0.087067385	0.038739852	0.105996539	Up
DIAPH3	1.045269104	0.063874411	0.040129279	0.109040938	Up
FOXC1	1.04188309	0.059193402	0.041122295	0.110973865	Up
KRT18	1.03314037	0.047036283	0.044671417	0.119731553	Up
ANXA2	1.055522024	0.077956682	0.046217137	0.123037513	Up
UCHL1	0.862101328	-0.214070647	4.40E-05	0.00474285	Down

Gene	FC	Log2fc	*P* value	*P* adj	Direction
BEX1	0.85822909	-0.220565293	4.45E-05	0.00474285	Down
STUB1	0.942850946	-0.08489838	4.81E-05	0.00474285	Down
CISD2	0.870710629	-0.19973476	5.54E-05	0.00474285	Down
PRKAA2	0.852061937	-0.23096979	6.07E-05	0.00474285	Down
PEBP1	0.93851584	-0.091547	0.000139052	0.00782662	Down
KRAS	0.925024838	-0.112435991	0.000167232	0.007846327	Down
EIF4H	0.956502643	-0.06415914	0.000179315	0.007846327	Down
STYK1	0.848564646	-0.236903522	0.000199145	0.007846327	Down
MYH10	0.894440535	-0.160942524	0.000320467	0.011478536	Down
ATP6V1E1	0.927487256	-0.108600637	0.00036684	0.012044581	Down
DDB1	0.945586317	-0.080718936	0.000442411	0.012135478	Down
RUFY3	0.940866426	-0.087938175	0.000473036	0.012135478	Down
CCT5	0.86387819	-0.211100194	0.000500171	0.012135478	Down
VAPA	0.909096085	-0.137495309	0.000506504	0.012135478	Down
SNCA	0.912398993	-0.13226324	0.000523612	0.012135478	Down
PGK1	0.927076105	-0.109240318	0.00056599	0.012388893	Down
PFN2	0.930739008	-0.103551422	0.000691615	0.012758111	Down
VAPB	0.938354855	-0.091794489	0.000698646	0.012758111	Down
WBP11	0.942179816	-0.085925668	0.000726487	0.012758111	Down

Gene	FC	Log2fc	*P* value	*P* adj	Direction
VDAC3	0.937046044	-0.093808156	0.000737957	0.012758111	Down
YWHAG	0.872037817	-0.197537394	0.000756576	0.012758111	Down
YME1L1	0.945667579	-0.080594958	0.000855049	0.012758111	Down
ATG16L1	0.951533192	-0.071674113	0.000893544	0.012758111	Down
RPL15	0.95891133	-0.060530678	0.000933315	0.012758111	Down
USP11	0.942273821	-0.085781732	0.000945765	0.012758111	Down
ATG5	0.917765127	-0.123803107	0.000952382	0.012758111	Down
LRPPRC	0.937612915	-0.092935653	0.000971132	0.012758111	Down
MDH2	0.946284473	-0.079654141	0.00097143	0.012758111	Down
XRCC6	0.955629484	-0.065476729	0.001132527	0.013944241	Down
IREB2	0.93486372	-0.097172024	0.001315234	0.014575156	Down
SETDB1	0.936786221	-0.09420824	0.001319455	0.014575156	Down
CISD1	0.916058235	-0.126488779	0.00133174	0.014575156	Down
ACVR1B	0.916455477	-0.125863301	0.001408622	0.014611975	Down
CNBP	0.965199443	-0.051101011	0.001490066	0.01505349	Down
CCT6A	0.935582246	-0.09606361	0.001595087	0.015331883	Down
RPA2	0.96387921	-0.053075731	0.001842497	0.017284376	Down
PSMA1	0.924597091	-0.113103272	0.002049287	0.018518828	Down
GPI	0.943385187	-0.084081147	0.002102638	0.018518828	Down

Gene	FC	Log2fc	*P* value	*P* adj	Direction
VDAC2	0.948369787	-0.076478392	0.002194639	0.018797562	Down
SIRT3	0.962675859	-0.054877983	0.002259473	0.018941117	Down
CLTB	0.924919926	-0.112599623	0.002393157	0.019242937	Down
ENO2	0.913063593	-0.131212751	0.002519569	0.019472662	Down
YWHAB	0.930390268	-0.104092089	0.00253096	0.019472662	Down
ACSL4	0.934774156	-0.097310247	0.002569996	0.019472662	Down
VDAC1	0.946368504	-0.079526034	0.002786363	0.020441874	Down
ASAH2	0.939367788	-0.090237972	0.002862427	0.020505386	Down
DLD	0.935791943	-0.095740288	0.002983282	0.020989519	Down
FH	0.962851275	-0.054615122	0.00377267	0.025628137	Down
MTOR	0.970962385	-0.042512688	0.003912081	0.026124744	Down
TTBK2	0.917381139	-0.124406848	0.003998774	0.026258619	Down
CUL4B	0.955936957	-0.065012618	0.004117142	0.026592686	Down
PSMD8	0.95821552	-0.061577915	0.005032944	0.03098406	Down
MEF2C	0.910878922	-0.134668797	0.005318524	0.03158066	Down
TPD52L2	0.954913031	-0.06655875	0.005370315	0.03158066	Down
SNRPD1	0.971395026	-0.041869995	0.005824777	0.032886758	Down
RHOT1	0.949682893	-0.074482229	0.005842825	0.032886758	Down
FBXW7	0.879619755	-0.18504809	0.006200364	0.033604854	Down

Gene	FC	Log2fc	*P* value	*P* adj	Direction
YWHAZ	0.953596895	-0.068548556	0.006296719	0.033604854	Down
GNB1	0.964421785	-0.052263855	0.006311571	0.033604854	Down
KEAP1	0.967829797	-0.047174738	0.006617398	0.03415185	Down
SRSF8	0.948333316	-0.076533875	0.006674346	0.03415185	Down
RBX1	0.967443862	-0.047750147	0.007046917	0.035595966	Down
XRCC5	0.97879597	-0.030919934	0.007620047	0.037065416	Down
NFS1	0.963490895	-0.053657062	0.008056705	0.038384218	Down
GOT1	0.968157774	-0.046685921	0.008086015	0.038384218	Down
NOLC1	0.915581086	-0.127240434	0.008203244	0.038477122	Down
KBTBD3	0.95164517	-0.071504343	0.008443999	0.039140418	Down
PTEN	0.950643238	-0.073024074	0.010239999	0.043853907	Down
DAZAP1	0.957620229	-0.062474467	0.010378138	0.043967593	Down
ELAVL1	0.966022172	-0.049871793	0.011462741	0.046084899	Down
BECN1	0.968208545	-0.046610268	0.011608943	0.046201249	Down
NAP1L	0.952475936	-0.07024545	0.014961312	0.057230651	Down
ABCB10	0.926967485	-0.10940936	0.015984502	0.060516607	Down
ACADSB	0.941563681	-0.086869423	0.016291093	0.060553684	Down
GPX4	0.967092469	-0.048274254	0.018242383	0.065992982	Down
CCT4	0.962676863	-0.054876478	0.018858873	0.066940504	Down

Gene	FC	Log2fc	*P* value	*P* adj	Direction
ARNTL	0.953112843	-0.069281064	0.019624314	0.069035533	Down
UBE2D3	0.952851562	-0.06967661	0.020069795	0.069977868	Down
CCT8	0.970419083	-0.043320175	0.022294206	0.075602267	Down
NEDD4L	0.94898558	-0.07554193	0.023450859	0.075602267	Down
CREB1	0.912643028	-0.131877421	0.023552513	0.075602267	Down
PANX1	0.94106503	-0.087633675	0.023601723	0.075602267	Down
MYCN	0.904946772	-0.144095157	0.026638907	0.08464298	Down
OTUB1	0.971325609	-0.041973096	0.027944787	0.088081969	Down
YTHDC2	0.900548824	-0.151123599	0.028248195	0.088298928	Down
AHCY	0.972571764	-0.040123387	0.028461837	0.088298928	Down
HNRNPM	0.96169298	-0.056351707	0.030491581	0.093137391	Down
GSK3B	0.972684647	-0.039955949	0.031600525	0.09570583	Down
KHDRBS1	0.965672767	-0.050393702	0.031820974	0.09570583	Down
DNAJB6	0.965975237	-0.049941889	0.032905066	0.098216636	Down
ABCB6	0.963712413	-0.053325407	0.03450516	0.10145547	Down
DUT	0.965058277	-0.05131203	0.035121027	0.101792286	Down
LDHA	0.951635609	-0.071518839	0.035136424	0.101792286	Down
PRC1	0.969274963	-0.04502211	0.035679188	0.102401225	Down
BAP1	0.966993628	-0.048421712	0.036049033	0.102401225	Down

Gene	FC	Log2fc	*P* value	*P* adj	Direction
EWSR1	0.976979379	-0.033599983	0.037145767	0.102578616	Down
ACSL3	0.948311884	-0.07656648	0.03723031	0.102578616	Down
SF3B1	0.97338314	-0.03892031	0.047112952	0.124580558	Down
YTHDF3	0.929093299	-0.106104616	0.047552777	0.124905295	Down
DPYSL2	0.962412945	-0.055272048	0.049551845	0.129294218	Down
